# Acknowledgment to Reviewers of *Vaccines* in 2020

**DOI:** 10.3390/vaccines9020089

**Published:** 2021-01-26

**Authors:** Vaccines Editorial Office

**Affiliations:** MDPI AG, St. Alban-Anlage 66, 4052 Basel, Switzerland

Peer review is the driving force of journal development, and reviewers are gatekeepers who ensure that *Vaccines* maintains its standards for the high quality of its published papers. Thanks to the cooperation of our reviewers, in 2020, the median time to first decision was 14 days and the median time to publication was 34 days. The editors would like to express their sincere gratitude to the following reviewers for their precious time and dedication, regardless of whether the papers were finally published:
Abate, GiuliaLuttermann, ChristineAbdallah, AliLykoudis, Panagis M.Abdel-Whab, El-SayedMabashi-Asazuma, HideakiAbouelhadid, SherifMachado, YoanAdunyah, Samuel E.Machelart, ArnaudAgallou, MariaMaciel, MiltonAgoro, RafiouMacNeill, AmyAkbar, SaminaMacRaild, ChristopherAkdesir, EzgiMaeda, AkihikoAkiyama, HisashiMagez, StefanAlaimo, AlessandroMagnano Di San Lio, GaetanoAlam, SaminaMahmood, IftekharAlbarnaz, J. D.Mahmoud, AymanAlbendín-García, LuisMahony, Timothy JohnAlbrecht, RandyMalejczyk, JacekAlcantara, Luiz Carlos JuniorMaltezou, HelenaAlderson, MarkManam, SrikanthAlejo, AliManavalan, BalachandranAlexander, LaiMancianti, FrancescaAli, Hayssam M.Mang, Stefania MirelaAllain, Jean-PierreMangala Prasad, VidyaAllegaert, KarelManini, IlariaAlmeida, Afonso Rocha MartinsMantzoukas, SpiridonALMOUAZEN, EyadManzoor, RashidAlsaggaf, RotanaMaple, PeterAltenburg, ArwenMaranesi, MargheritaAltmann, DannyMarc, DanielAlvarez, F. JavierMarć, Małgorzata AnnaAlzan, HebaMarchiò, CaterinaAMAROWICZ, RyszardMarenzoni, Maria LuisaAmiel, EyalMarin Lopez, AlejandroAmnie, AsratMarkham, RichardAndreev, KonstantinMaro, JudithAndreoni, FrancescaMarracci, SilviaAndreuzzi, EvaMartenot, ClaireAngelillo, ItaloMartin, JessicaAngius, FabrizioMartin, NeginAnstead, Gregory M.Martín, VeronicaAnticoli, SimonaMartín-Acebes, Miguel A.Antonelli, AlbertoMartinez, OsvaldoAntony, AntoniouMartin-Galiano, Antonio J.Antonyuk, SvetlanaMartini, CeciliaAntwi, Samuel O.Martins, IanAnushree Seth, AnushreeMathieu, CyrilleApostolopoulos, VassoMattner, JochenAppavu, RajagopalMattoscio, DomenicoArgentiero, AntonellaMaurya, ShailendraAriel, AmiramMaynard, JenniferArora, GunjanMazrier, H.Arruda, Bailey L.McCarthy, PumtiwittAsfor, AminMcIntosh, E. David G.Asghar, ZahidMcKinstry, Karl KaiAtanasova, KalinaMcLean, GaryAttia, YoussefMcMahon, MeaganAudet, JonathanMcMichael, LeeAurora, RajeevMcMurray, David N.Awasthi, MayankaMcVey, David ScottAwasthi, SitaMedina, Gisselle N.Azab, WalidMehariya, SanjeetAzuma, ChiekoMei, Kuo-ChingBabichev, SergiiMei, YangBabiuk, ShawnMeier, Jeffery L.Bachmann, MartinMeli, VijaykumarBagiu, Iulia-CristinaMelo, PedroBaigent, SueMercer, AndyBailly, BenjaminMereu, AlessandraBaindara, PiyushMerino, Jose JoaquinBakre, Abhijeet A.Mesalam, AhmedBalan, SreeKumarMeseda, ClementBalasch, MonicaMesquita, JoãoBaldanzi, GianlucaMestres, ConxitaBall, Patrick A.Metzger, Herr PhilippBallegeer, MarliesMetzger, MichaelBamford, ConnorMeurens, FrancoisBan, BhupalMeurens, FrançoisBanadyga, LoganMeyn, LeslieBansal, AnjuMicheau, OlivierBarb, Adam WMihala, GaborBarbezange, CyrilMiki, YasuhiroBarbosa, Raquel De MeloMilavetz, BarryBarcena, JuanMiller, Kenneth E.Bardi, GiuseppeMinami, MasaakiBarik, SailenMinervini, Crescenzio FrancescoBarr, Kelli L.Miranda-Sanchez, FabiolaBarra, GiusiMishto, MicheleBarrow, Alexander DavidMisra, Ravi SBartholomeeusen, KoenMitsios, AlexandrosBartok, EvaMittler, Eva-MariaBarton, ChrisMiyazawa, MasaakiBashford-Rogers, RachaelMizukami, ShusakuBashkin, James K.Mlaga, Kodjovi D.Batchu, RameshMlera, LuwanikaBates, John T.Moccia, MarcelloBattah, BasemMohammed, AltafBawage, SwapnilMoiré, NathalieBaxter, Victoria K.Mojumdar, KamalikaBayer, WibkeMolinier-Frenkel, ValérieBeauvais, Wendy A.Mondal, SomritaBeckham, J. DavidMondal, Utpal KumarBednarczyk, Robert A.Monroy, FernandoBehrendt, PatrickMontoya, AnaBeissert, TimMorales Suárez-Varela, María M.Bellini, SilviaMoreland, NikkiBenati, DanielaMoren, AlainBendahmane, MohammedMorgan, EthanBenfield, David A.Morgan, Ethan L.Benmerzouga, ImaanMoricoli, DiegoBennett, RichMorikawa, YukoBentel, Jacqueline M.Moro, Pedro L.Berentsen, AreMorozov, IgorBerger, FabianMorris, Christopher JBerges, Bradford K.Moss, BernardBerkes, CharlotteMoss, WalterBernardino, Raquel L.Mossel, EricBernatchez, Jean A.Motaze, VillyenBernstein, Henry H.Motin, VladimirBertelloni, FabrizioMotwani, MonaBertram, MirandaMousa, Jarrod J.Bessaud, MaëlMu, QinghuiBettencourt, PauloMukherjee, RatnadeepBharaj, PreetiMukherjee, SomnathBhattacharya, AbhisekMukherjee, SumitBhide, YoshitaMukundan, SanthoshBianco, FrancescoMüller, UweBianco, GiulianaMunang’andu, Hetron MweembaBicout, DominiqueMunderloh, UrlikeBielefeldt-Ohmann, HelleMurata, ShiroBins, Adriaan D.Murdaca, GiuseppeBirlea, MariusMuthu Karuppan, Mohan KumarBlaise-Boisseau, SandraMuthuramu, IlayarajaBlanco-Fernandez, BarbaraMutinelli, FrancoBobbala, SharanMuttil, PavanBodomo, AdamsMuxel, Sandra MarciaBoggio, ElenaMyc, AndrzejBøgwald, JarlMymryk, JoeBonam, Srinivasa ReddyN. Medina, GisselleBonavita, EduardoNachbagauer, RaffaelBordonaro, MichaelNagasaka, KazunoriBorena, Wegene T.Nagata, ToshiBorsetti, AlessandraNaguib, MahmoudBorza, TudorNakamura, KyoheiBosco-Lauth, Angela M.Nakanishi, AkiraBose, TanimaNakatani, YoshihikoBourret, VincentNakayama, TetsuoBowen, AnthonyNakayasu, ErnestoBowen, RichardNanduri, RavikanthBradfute, StevenNanjappa, Som G.Brai, AnnalauraNarayan, EdwardBraicu, CorneliaNarayan, Ram NarendraBranca, Jacopo Junio ValerioNarayanan, MuthuBriana, Despina D.Narra, Hema PrasadBriles, David E.Narwani, Tarun JaiRajBriza, PeterNashar, TouficBrocchi, EmilianaNasti, Tahseen HBrokstad, Karl AlbertNazki, SalikBrown, MarkNde, Pius N.Brown, Richard J.P.Neerukonda, SabarinathBrown, Ronald B.Nepal, Mahesh RajBrunner-Weinzierl, Monika ChristineNeter, EfratBruschi, FabrizioNeuman, BenjaminBryan S., KaplanNeureiter, DanielBugarin, AlejandroNg, Qin XiangBull, RowenaNghia, Truong PhuocBurckhardt, ChristophNguyen, Tan B.Burgert, HansNgwa, Che JuliusBurrascano, JosephNicoletti, LoredanaBurrello, JacopoNiederwieser, DietgerBush, Stephen J.Niikura, MasahiroBuxton, SamuelNitta, TakayukiByadgi, Omkar VijayNocentini, GiuseppeCaballero, PabloNociari, MarceloCalapai, GioacchinoNogales, AitorCalistri, AriannaNogueira, PauloCampana, StefaniaNowalk, Mary PatriciaCampos, Samuel K.Nugent, KennethCandéias, SergeNúñez, José IgnacioCaneiras, CátiaNuttall, Patricia ACantos-Barreda, AnaNzelu, ChukwunonsoCanuti, MartaO’Connor, DanielCao, ChuanhaiOdland, JonCao, DianjunOgasawara, NorikoCapucci, LorenzoOgino, ShujiCara, AndreaOgunjirin, AdebowaleCardeti, GiusyOhkuri, TakayukiCarey, AlisonOkamoto, ShigefumiCaroleo, Maria CristinaOliver, JoAnneCarow, BeritOomens, Antonius (Tom)Carrillo, ConsueloOpata, MichaelCary, LynnetteOrnelles, DavidCasares, NoeliaOrtega, EduardoCastiello, LucianoOsorio, Elvia JanethCastillo, JulioOthumpangat, SreekumarCastro-Lopez, NataliaOtlewski, JacekCavalletto, LuisaOtsuka, IsaoCenciarelli, CarloOttmann, MichèleCerón-Carrasco, José PedroOzen, MehmetCerwenka, HerwigPacenti, MoniaChackerian, BrycePaeshuyse, JanChaganty, BharatPahar, BapiChakrabarti, RajarshiPaik, Soon YoungChambers, ThomasPain, BertrandChand, SubhashPainter, HeatherChandrashekar, AbishekPali-Schöll, IsabellaChang, Che-ChienPaolini, FrancescaChang, Kwang PooPapoutsoglou, PanagiotisChao, Chien-ChungPapp, BélaChao, K.S. CliffordParan, NirChatterjee, NimratParida, SatyaChatterjee, SatabdiParikesit, Arli AdityaChattopadhyay, SaurabhParisini, EmilioChaves, ElenaPark, EnochChaves, Luis FernandoPark, Se ChangChavez-Calvillo, GabrielaParvez, MohammadChen, Hui-WenPatching, Simon GChen, LiliPathak, DhrubaChen, TingtingPavelko, Kevin D.Chen, Yu-ChunPawelec, GrahamChen, ZhongPazgier, MarzenaChen, ZiqingPeckham, Gabriel D.Cheng, HaoPeeples, MarkCheng, ZishuoPei, JingjingCheshenko, NataliaPei, YonggangChia, GlorynPeinemann, FrankChiang, Yi-TingPejin, BorisChiara, FocaccettiPejoski, DavidChlichlia, KaterinaPence, BrandtChoi, Won HyungPeng, DuoChoi, Youkyung H.Pennock, NathanChowdhury, Imran HussainPenrith, Mary-LouiseChowdhury, RatulPentakota, Sri RamChristodoulides, MyronPépin, MichelChung, Hee-ChunPeran, IvanaChung-Davidson, Yu-WenPerdomo, JoseCipak, LubosPérez-Martínez, ClaudiaCipolloni, LuigiPérez-Sánchez, RicardoClanchy, FelixPeriago, Maria VictoriaCloninger, MaryPerišić, OgnjenCocco, RaffaellaPersano, StefanoCocuzza, ClementinaPeruzzi, FrancescaColditz, IanPeters, ChristianComas-Garcia, MauricioPetousis-Harris, HelenComer, Jason E.Petravić, JankaCompton, AlexPetrovan, VladConlan, WaynePetrovski, KiroConway, BrianPeyton, David H.Cook, CharlesPezzoni, GiuliaCooke, BrianPhan, TungCoombs, KevinPiantino Ferreira, Antonio JoséCoombs, Kevin MPiatkevich, KirylCoppeta, LucaPichon, MaximeCorreia Coelho, Ana CláudiaPickering, RaeleneCosby, LouisePiercy, HilaryCosco, DonatoPilones, Karsten AndersonCosseddu, Gian MarioPimenoff, Ville N.Crauste, FabienPincus, SethCrawford, LindseyPineau, PascalCrisi, Paolo EmidioPiñeiro, LuisCrnkovic, AnaPinheiro, JorgeCrook, BrianPiotrowski, PaulinaCwynar, PrzemyslawPiscitelli, PriscoD’Apice, LucianaPistello, MauroDacosta, Miguel BaladoPittau, MarcoDahiya, SatinderPiu, SahaDahl, Jan-UlrikPizzorno, Mario AndresDai, Chia-YenPlant, EwaDal Col, JessicaPlaza-Sirvent, CarlosDalmo, RoyPloquin, AurelieDamiaans, BertPoddighe, DimitriD’Ancona, Fortunato PaoloPolano, MaurizioDandekar, AdityaPoncet, DidierDas, AnshumanPontejo, Sergio MDas, RupaliPoole, Brian D.Datan, EmmanuelPossee, RobertDatta, RupsaPotaczek, Daniel P.Davidson, IritPoulet, HervéDavis, April D.Prasad, AbhishekDawen Yu, EstherPrearo, MarinoDawood, Mahmoud A.O.Preziuso, SilviaDe Bernardi Schneider, AdrianoPrice, PatriciaDe Jesus Pereira Godinho Velada, IsabelPrincipe, Daniel R.De Robertis, MariangelaPrinz, ImmoDe Rojas, ManuelPuig-Barberà, JuanDe Sanctis, FrancescoPuolakkainen, MirjaDe Sanctis, Juan BautistaPyo, Hyun MiDea-Ayuela, MaríaPyzik, MichalDean, MichaelQi, JunpengDebin, TianQi, YanfeiDeim, ZoltánQiu, Hua-JiDel Fresno Sánchez, CarlosQuan, Fu-ShiDel Mistro, AnnarosaQuan, Fu-ShuiDel Real, GustavoQuesada, ErnestoDelhon, GustavoRaeven, RenéDelitala, AlessandroRaffray, LoicDell’Oste, ValentinaRafi, JunaidDelmonico, LucasRajagopal, SudarshanDeLong, GayleRajko-Nenow, PaulinaDelputte, PeterRaju, NagarajanDesselberger, UlrichRakotondrafara, AurelieDettori, MarcoRamaiahgari, Sreenivasa C.Dhakal, SantoshRamchandani, DivyaDhandayuthapani, SubramanianRanjan, AmareshDi Foggia, MicheleRanstam, JonasDi Gioia, MarcoRappo, UraniaDi Giovanni, PamelaRappoport, NadavDi Martino, GiuseppeRappuoli, RinoDi Profio, FedericaRascher, WolfgangDi Sotto, AntonellaRather, IrfanDiaz De Cerio, OihaneRattanapan, CheerawitDiaz-San Segundo, FaynaRavenscroft, NeilDiefenbach, Russell J.Ravichandran, SupriyaDiehl, WilliamRay, Heather J.Dima, AlinaRay, SamriddhaDimitrov, KirilRazzuoli, ElisabettaDing, JianxunReagan, John LDING, LingwenReeves, MatthewDixit, SurjitRegunath, HariharanDixon, BrianReid, ScottDolan, Brian P.Reid, St PatrickDomenech, AnaReif, Kathryn E.Domnich, AlexanderReifinger, MartinDonadu, Matthew GavinoReiger, MatthiasDonald, Claire L.Reina, RamsésDonham, KelleyReis, Alexandre BarbosaDoody, GinaReis, AnaDosoky, NouraRen, ZhenhuaDowgier, GiuliaRenu, SankarDragomir, MihneaResa-Infante, PatriciaDrillien, RobertReshkin, Stephan JoelDu, JuanRety, StephaneDubois, JuliaRevilla, YolandaDucatez, MarietteRey, DavidDuerr, ClaudiaReyes-del Valle, JorgeDuhan, VikasReyes-López, Felipe E.Duizer, ErwinRibi, CamilloDuncan, SteveRiccò, MatteoDundon, WIlliamRichetta, ClémenceDunowska, MagdalenaRichner, JustinDürrwald, RalfRichner, Justin M.Dzięgiel, PiotrRigby, MichaelDziejman, MichelleRissanen, IlonaEbensperger, GermánRiva, AntonioEcarnot, FionaRivera-Toledo, EvelynEda, ShigetoshiRizk, Mohamed AbdoEdlefsen, Paul T.Roberto Da Silva, EdsonEhrhardt, AnjaRobinson, Sally R.Ehtisham-ul-Haque, SyedRoby, Justin A.Eldeeb, MohamedRockman, StevenEldridge, SandyRodrigues, Célia F.Elena, CriscuoloRodriguez Valle, ManuelEl-Gazzar, Mohamed M.Rodríguez Villanueva, JavierEmbers, MonicaRodriguez, Gloria MarcelaEngelhardt, OthmarRoedel, FranzEngland, MarionRolla, SimonaErrington-Mais, FionaRomano, FerdinandoEschbaumer, MichaelRomano, MariaEscudero-Pérez, BeatrizRonca, ShannonEspel, EnricRonsard, LaranceEssani, KarimRoque, AliciaEverest, PaulRosa, Bruce A.Fairchild, GeoffreyRosato, EdoardoFang, Ronnie H.Rosenke, KyleFaraone Mennella, Maria RosariaRossi, FrancaFarnos, OmarRossi, OmarFarrell, HelenRossman, JeremyFaviana, PinucciaRothan, HussinFeás, XesúsRotondo, John CahrlesFeldman, SteveRouth, AndrewFeng, NingguoRouthu, Nanda KishoreFernández, Ivan PastorRuaro, BarbaraFerrante, ClaudioRuiz Téllez, TrinidadFerrara, FrancescaRussell, TiffanyFerrari, PaolaRusso, RiccardoFerreira, FernandoRyabov, EugeneFerreira, Helena LageRybka, JakubFerreira, Leonardo M.R.Rybka, Jakub DaliborFichera, EpifanioRyter, KendalFielding, CeriRyu, Keun HoFisher, ScottRzymowska, JolantaFitton, HelenSachithanandham, JaiprasathFleming, JohnSadigh, YasharFletcher, Nicola F.Sahani, RajkumarFlint, MikeSahlin, UllrikaFlorek, EwaSalak-Johnson, JaneenFonseca, FilomenaSalpini, RominaFonseca, KevinSamhan-Arias, AlejandroFontana, JuanSamir, ParimalForlenza, MariaSampaio, Natalia G.Forth, Jan HendrikSamy, AbdallahFossum, EvenSamy, AhmedFoster, Toshana LauriaSanchez Vargas, Luis AlbertoFox, JulieSanchez, David JesseFracasso, GiulioSanchez, Maria TFrade, João Manuel GraçaSanders, NiekFranco-Molina, Moises ArmidesSanftenberg, LindaFranzoni, GiuliaSang, YongmingFraser, JohannaSangster, Mark Y.Fratini, FilippoSantamera, AntonioFreuling, ConradSantos, JeffersonFrias Casas, MarioSanyal, MrinmoyFrossard, Jean-PierreSarathy, Vanessa V.Fry, LindsaySardu, CelestinoFuery, AngelaSarkar, AmritaFuller, Frederick J.Sarker, SubirFuruse, YukiSartini, DavideFuruya, TetsuyaSasaki, HirakuFuruya, YoichiSatheshkumar, Panayampalli SubbianGabrieli, PaoloSatish, LakkakulaGacesa, RankoSato, HiroshiGala, Rikhav P.Satoshi Kashiwagi, SatoshiGalindo-Villegas, JorgeSauder, ChristianGambarian, AlexandraSaurav, KumarGanaie, SafderSauter-Louis, CarolaGao, MinSautto, Giuseppe A.García-Sánchez, SusanaSavelyeva, NataliaGatto, FrancescaSawa, TeijiGaudreault, Natasha NSaxena, PoojaGauld, Natalie JoanSaxena, VikasGautier-Bouchardon, Anne-V.Schafer, Katherine MontagGazerani, ParisaSchlaepfer, Isabel RGehring, ChristophSchlecht-Louf, GéraldineGenovese, CristinaSchlegel, Peter N.Georgel, PhilippeSchmitt, ArminGershwin, LaurelSchmolke, MircoGesesew, Hailay AbrhaSchoen, Robert T.Ghag, GauravSchrama, DeniseGhildyal, ReenaSchreiber, MichaelGhosh, SumitSchulte, JoannGianfredi, VincenzaSchusser, Gerald FritzGiangaspero, MassimoSchuurman, Henk-JanGiannella, LucaSchwartz, David A.Gianola, SilviaSchwermer, HeinzpeterGimple, Ryan C.Scomparin, AnnaGirard, MarcScott, DanielGnanamony, ManuScotti, MarcusGnanasundram, Sivakumar VadivelSeed, SheilaGoh, Gerard Kian-MengSeferovic, Maxim D.Golde, William T.Seidl, MaximilianGolomb, BeatriceSekar, ShobanaGolubovskaya, VitaSeminari, ElenaGomes, Ana CordeiroSenel, MakbuleGomez Del Moral, ManuelSeo, Ho SeongGomez, Carmen ElenaSeo, Sang HeuiGomez-Casado, EduardoSeo, Young-JinGómez-Muñoz, María TeresaSepuru, Krishna MohanGonçalves, LídiaSergeeva, Oksana A.Gondek, MichałSerrano, IreneGonzales, Jose LSerwer, PhilipGonzalez, GabrielSeth, AnushreeGonzalez, Martin VillalbaSethi, GautamGoodier, Martin R.Sethi, Manveen K.Goodman, Alan G.Sevvana, MadhumatiGordon, David M.Shackelford, Rodney E.Gori, DavideShafer, Leigh AnneGórska, SabinaShah, HemangiGoto, TaichiroShakya, AkhileshGould, PhillipShakya, Viplendra Pratap SinghGoyvaerts, CleoShamputa, Isdore CholaGrabowski, JeffreySharma, ArvindGranero-Molina, JoseSharma, AtulGranito, AlessandroSharshov, KirillGraves, StephenSheikh, AlaullahGreim, HelmutShen, Ming-YiGrennan, TroySheu, JimGris, DenisShibuya, HiroshiGrose, CharlesShienfeld, YehudaGrubman, MarvinShimada, MasaruGruters, Rob A.Shimizu, TakeyukiGuardado-Calvo, PabloShimodaira, ShigetakaGuidolin, DiegoShin, Kwang-SooGuimarães, Jorge A.Shingai, MasashiGuito, JonathanShirai, JyunsukeGulic, TamaraShivanna, VinayGundamaraju, RohitShoemaker, CharlesGupta, KuldeepShridhar, SurekhaGustin, KurtShulman, Lester M.Hai, RongSiddharthan, VenkatramanHairgrove, ThomasSiegel, Sue RutherfordHåkansson, AndersSiettos, Constantinos I.Hall, Roy A.Signorelli, CarloHamiel, UriSil, PayelHammer, AnneSiltz, Lauren A.Hammerl, Jens AndreSilva, CatarinaHann, Hie-WonSilva, Marcelo SousaHao, MingangSimecka, Jerry W.Haqshenas, GholamrezaSinganalur, Nagendrakumar BalasubramanianHarada, MamoruSingh, Amit K.Hariharan, SeethalakshmiSingh, AnandHarrison, Paul H. M.Singh, AnupHart, GeoffreySingh, Brajendra KumarHarvill, EricSingh, ChanpreetHatfull, GrahamSingh, ReemaHause, BenSingh, ShaktiHauser, PeterSinha, DebottamHawkes, DavidSioofy-Khojine, Amir-BabakHayashi, RyujiSiozopoulou, VasilikiHayashi, TomokoSiristatidis, CharalamposHayes, Sidney J.Sivakumar, SushamaHays, John P.Skundric, Dusanka S.He, ChunboSkwarczynski, MariuszHe, JieSlavcev, RoderickHe, Jin-ShengSlütter, BramHe, MingyuSmith, JessicaHealey, GarethSmith, RichardHegde, ShivanandSmith, Tara C.Hegewald, JaniceSmith, Todd G.Heo, JeongSöderhäll, KennethHernandez, RaquelSolimando, Antonio GiovanniHerndler-Brandstetter, DietmarSong, Min-SukHerold, BetsySoni, ChetnaHerrick, Jesica AllynSounni, Nor EddineHersey, PeterSousa, AngelaHersperger, Adam R.Souza, PedroHIjikata, AtsushiSozhamannan, ShanmugaHildreth, EasonSpatz, StephenHinkula, JormaSpencer, Juliet V.Hippe, BeritSpiro, David J.Hirano, TsunahikoSridhar, SiddharthHirsch, IvanStanley, Jone A.Ho, Chak-SumStanton, RichardHo, Paulo LeeStefanoff, PawelHoff, Nicole AStehlík, MilanHoffmann, Klaus H.Steinhagen, DieterHofstetter, AmeliaStendahl, OlleHogenEsch, HarmStepanova, EkaterinaHojjat-Farsangi, MohammadStern, DanielHolbrook, MichaelStervbo, UlrikHolland, MartinStewart, AdamHollingsworth, DéirdreStoecker, CharlesHomem-de-Mello, MauricioStone, WendyHonda-Okubo, YoshikazuStout-Delgado, HeatherHong, XupengStrandin, TomasHooper, Douglas C.Strive, TanjaHorstman, KlasienStrohfus, Pamela K.Hort, KrishnaStromberg, ZacharyHoshina, TakayukiStrutt, Tara M.Hozbor, DanielaStutz, BernardoHsiao, Hui-HuaSu, Dong-MingHu, JiafenSuárez, Cinta PrietoHua, XiaoqingSubbian, SelvakumarHuang, ChengSubrata, SabuiHuang, JianshengSui, YongjunHuang, KaiSun, HaiyanHuang, TimothySun, KunfengHuang, XiaohuSun, WeinaHuang, Yan-JangSun, XiaoqiangHuang, YuSundberg, Eric J.Huang, YuanyuSung, Pil SooHuber, MichaelSuperti, FabianaHuber, VictorSuter, AndyHuman, StaceySutoh, YoichiHutcheson, H. JoelSuzutani, TatsuoHutchison, Robert E.Sweeney, TrevorHwang, Eung-SooTada, RuiI. Nistor, GabrielTagad, Harichandra D.Iampietro, MathieuTakahashi-Omoe, HiromiIbrahim, AhmedTAKAM KAMGA, PaulIchinohe, TakeshiTakano, TomomiIde, KazukiTalukder, PoulamiIlyinskii, Petr O.Tam, Roger Y.Imai, TakashiTamaki, NobuharuImamura, MasahiroTamayo, EstherInoue, JunTamsyn, StanboroughInoue, TakakoTan, Bee KInyushin, Mikhail Y.Tanaka, YoshikazuIorio, Ronald M.Tang, DamuIovane, AndreTang, JingjingIqbal, Hafiz M. N.Tang, QiyiIsakova-Sivak, IrinaTarang, ShikhaIshikawa, TomohiroTarasov, AndreiIslam, ArifulTardivo, StefanoIsmael, M. IsabelTarradas Font, JoanIto, NaotoTartey, SarangIwata, TakashiTauriello, DanieleIyer, HariniTaus, NaomiJackson, Sarah E.Tavakoli, Norma P.Jagodzinski, FilipTaylor, AdamJain, PrashantTaylor, Erika A.Jain, VaibhavTaylor, MatthewJangra, RohitTedbury, PhilipJanowicz, AnnaTejedor, SantiagoJanuszkiewicz-Lewandowska, DanutaTellier, MichaelJayaprakash, PriyamvadaTerryn, SanneJeannot, EmilienThaker, Youg RajJibran, RubinaThakur, AneeshJiménez-Barbero, JesusThanki, Anisha MahendraJin, TengchuanThiruvengadam, MuthuJohnson, Dylan M.Thomas, Stephen JJohnson, NicholasThomas, WayneJohnson, R. JeremyTinker, JulietteJohnson, Reed F.Tippett, VivienneJones, ChrisTiriveedhi, VenkataswarupJones, IanTischler, DirkJones, Rodney P.Tiwari, PoojaJoshi, KamalTodt, DanielJoshi, Shivali S.Toietta, GabrieleJost, StephanieTokars, Valerie L.Jung, Tae SungTomaszewska, EwaKaelber, JasonTonelli, MicheleKajihara, MasahiroTong, JessicaKalmbach, LotharTorras, JuanKamitani, ShigekiTortorella, DomenicoKammerer, RobertToth, ZsoltKanada, MasamitsuTraw, BrianKanda, NaokoTremblay, NicolasKanda, TatsuoTrevisan, AndreaKang, CongBaoTripathi, Jitendra KumarKang, Sang-MooTripp, LianneKanginakudru, SriramanaTrollfors, BirgerKanoi, BernardTruong, Nghia P.Kant, SashiTrus, IvanKao, Yu-HsiangTso, For YueKaplan, ShaunaTsukamoto, TetsuoKar, ShantimoyTsukiyama-Kohara, KyokoKarlsson, ErikTuells, JoséKarmakar, ParthaTufarelli, VincenzoKarniely, SharonTuma, RomanKarniychuk, UladzimirTun, Mya Myat NgweKarsten, Christina BeatriceTuomanen, ElaineKaruppannan, Anbu KumarTyliszczak, BożenaKatneni, UpendraUllah, IrfanKatsafadou, AngelikiUnterweger, ChristineKatsanos, Aristeidis H.Upadhyay, ChitraKatuchova, JanaUrra, José MiguelKatzenstein, TerezeUthaman, SajiKaufer, Benedikt B.Uversky, VladimirKawakami, JunjiV. GRAHAM, SheilaKawakami, MasanoriValenzuela, RodrigoKawano, KouichiroValkenburg, Sophie A.Kehn-Hall, KyleneValm, Alex M.Keller, FriederVan De Vijver, David AMCKenney, Joan L.Van Der Sluis, Renée M.Kesari, Kavindra KumarVan Dijk, Jitse P.Kesharwani, SiddharthVan Dyke, MichaelKesherwani, Varun KumarVan Gool, Stefaan W.Khalid, HattafVan Hooste, Wim Leo CelinaKhan, KrishnenduVan Muiswinkel, Willem B.Khan, NaeemVan Santen, VickyKhatami, AmenehVan Wagoner, Nicholas J.Khatri, VishalVanham, GuidoKhatun, AminaVann, WillieKhayat, RezaVaradaraj, ArchanaKhazandi, ManouchehrVarani, LucaKhuder, Sadik A.Varsani, ArvindKichik, NessimVasile, FrancescaKieber-Emmons, ThomasVasudevan, AbhinavKim, Han WoolVeeramani, SureshKim, JarimVendetti, SilviaKim, Jong GugVenditto, VincentKim, MeehyeinVenkatesan, ThiagarajanKim, Ryong NamVenuti, AldoKim, SunheeVerhoeven, DavidKimpel, JanineVerjans, Georges M.G.M.Kindrachuk, JasonVerli, HugoKipar, AnjiVersteeg, LeroyKirchner, SebastianVervelde, LonnekeKirkland, PeterVetrano, Ignazio GaspareKiseleva, Irina V.Víglaský, ViktorKishida, YutakaVignier, NicolasKishore, Dr. UdayVijayan, MadhuvanthiKlapper, PaulVillanueva, Rafael-JacintoKo, Eun-juVimercati, AntonellaKo, Hae-JinVital, Wendel CouraKobayashi, NobumichiVitale, FabrizioKobayashi, TetsuroViviani, SimonettaKobeissy, FirasVögtlin, AndreaKobiler, OrenVoliani, ValerioKoh, HyunwookVos, AdKOICHI, HASHIMOTOVoshavar, ChandrashekharKolawole, AbimbolaVoss, MatthiasKonjufca, VjollcaWagner, AbramKosik, IvanWald, AnnaKovbasnjuk, OlgaWang, Ching-HoKozieł, EdmundWang, DanKozlov, AndreyWang, JunKrauss, Isaac J.Wang, KaiKreys, EugeneWang, LeyiKroeker, AndreaWang, LinKrol, EwelinaWang, NianshuangKrzton, AliWang, PenghuaKshirsagar, Prakash G.Wang, Sheng-FanKubo, TaiWang, XiKukshal, VandnaWang, YouxinKulkarni, RaviWAYE, Mary M.Y.Kumar, BinodWebley, WilmoreKumar, GokhleshWei, HongpingKumar, HirdeshWeir, DawnKumar, RameshWeissenhorn, WinfriedKumaraswamy, ChitralaWelch, SteveKumari, AshaWelliver, RobertKunoh, TatsukiWen, ZhiweiKuroda, DaisukeWertz, Philip W.Kwilas, StevenWestra, JohannaKwon, Hyuk-JoonWibowo, DavidKyei, George BWieckowski, SébastienL. Schmaljohn, AlanWigfall, LisaLa Torre, GiuseppeWiley, Clayton A.Lacasta, AnnaWilliams, DianaLahooti, HooshangWilliams, E. MarkLai, AlexanderWilliams, GrahamLai, Zon WengWilliamson, E. D.Lamichhane, PurushottamWilm, MatthiasLandolt, Gabriele A.Wilson, Jo LeanneLang, FengchaoWilson, Van G.Lange, Margaret J.Wilson, WilliamLangereis, Jeroen D.Witkowski, WojciechLangosch, DieterWittmann, JürgenLangsjoen, RoseWohlgemuth, NicholasLanteri, GiovanniWong, Ching-OnLanza, GiuseppeWong, Pamela T.Laparra, Jose MoisésWorch, RemigiuszLapuente, DennisWozniak-Knopp, GordanaLarrous, FlorenceWu, Jack H.Z.Lasecka-Dykes, LidiaWu, Suh-ChinLavanchy, DanielWu, XianfangLavender, KerryWu, XuelingLe Doaré, KirstyXia, NingshaoLe Moigne, VincentXia, YuchenLe, Hoang-ThanhXiao, LiLeclerc, DenisXiao, YongliLecollinet, SylvieXie, HangLecureux, JonathanXie, XupingLedder, GlennXu, JianLedgerwood, Emily DesmetXu, YifeiLee, ChoonghoXu, ZhiweiLee, Heung KyuXue, GuangaiLee, Kyung-WooYager, EricLee, YoungYAKAVETS, IlyaLehman, John M.Yamada, HiroyukiLemiere, StephaneYamanaka, AtsushiLeng, SiyangYamauchi, AkiraLeroux, Louis-PhilippeYamayoshi, SeiyaL’homme, LaurentYan, Hongbin (Tony)Li, ChunfengYang, BoLi, DongmeiYang, LiliLi, LiYang, Yi-YuanLi, Mei-LingYaron, Jordan R.Li, Shengwen CalvinYoko, MatsuzakiLi, ShitaoYokoyama, ShinjiLi, WenboYoo, So YoungLi, XiaojunYoon, Sun-WooLi, YingYouk, SungsuLichner, ZsuzsannaYoung, Sean G.Liechti, George W.Yu, Chia-LiLillini, RobertoYu, JingyouLin, HaishuYu, QingzhongLin, Hung-YinYura, YoshiakiLin, QishanZakhartchouk, AlexanderLin, TaoZeeb, HajoLin, YaoZell, RolandLindhout, DickZella, DavideLinial, MichalZha, ChenLippert, ElisabethZhan, BinLIU, FANGZhang, GuofangLiu, GuanQunZhang, JinyangLiu, HaipengZhang, PengLiu, HaolinZhang, QingrunLiu, MargaretZhang, RuiLiu, Shih-JenZhang, ShuoLiu, XiaoyingZhang, XiangminLizano, MarcelaZhang, XinxingLo Duca, AngelicaZhang, YijunLo Giudice, RobertoZhang, YinfengLoncoman, CarlosZhang, YuningLong, QuanZhao, LinboLopez-Canul, MarthaZhao, QinjianLópez-Sanmartín, MonserratZhao, YangLópez-Siles, MireiaZheng, WenshuŁopusiewicz, ŁukaszZhong, ZhaohuaLorberboum-Galski, HayaZhou, DongmingLoring, Ralph H.Zhou, FanLu, WenchaoZhu, LuchangLucchese, GuglielmoZhu, XueyongLuce-Fedrow, AlisonZhuang, XiaoxuanLuciana, RossiZienius, DainiusLuciani, MirellaZimmer, GertLukashevich, IgorZiora, Zyta MariaLukyanov, Pavel A.Zou, ChunbinLunge, Vagner RicardoZou, WeiLuthra, PriyaZowalaty, Mohamed E. ElLutter, ErikaZumbrun, Elizabeth

